# Neutral genetic variation in adult Chinook salmon (*Oncorhynchus tshawytscha*) affects brain-to-body trade-off and brain laterality

**DOI:** 10.1098/rsos.170989

**Published:** 2017-12-06

**Authors:** Mallory L. Wiper, Sarah J. Lehnert, Daniel D. Heath, Dennis M. Higgs

**Affiliations:** 1 Department of Biological Sciences, University of Windsor, Windsor, Ontario, Canada N9B 3P4; 2 Great Lakes Institute for Environmental Research, University of Windsor, Windsor, Ontario, Canada N9B 3P4

**Keywords:** somatic trade-off, energy trade-off hypothesis, laterality, brain lateralization, inbreeding, heterozygosity

## Abstract

Low levels of heterozygosity can have detrimental effects on life history and growth characteristics of organisms but more subtle effects such as those on trade-offs of expensive tissues and morphological laterality, especially of the brain, have not been explicitly tested. The objective of the current study was to investigate how estimated differences in heterozygosity may potentially affect brain-to-body trade-offs and to explore how these heterozygosity differences may affect differential brain growth, focusing on directional asymmetry in adult Chinook salmon (*Oncorhynchus tshawytscha*) using the laterality and absolute laterality indices. Level of inbreeding was estimated as mean microsatellite heterozygosity resulting in four ‘inbreeding level groups’ (Very High, High, Medium, Low). A higher inbreeding level corresponded with a decreased brain-to-body ratio, thus a decrease in investment in brain tissue, and also showed a decrease in the laterality index for the cerebellum, where the left hemisphere was larger than the right across all groups. These results begin to show the role that differences in heterozygosity may play in differential tissue investment and in morphological laterality, and may be useful in two ways. Firstly, the results may be valuable for restocking programmes that wish to emphasize brain or body growth when crossing adults to generate individuals for release, as we show that genetic variation does affect these trade-offs. Secondly, this study is one of the first examinations to test the hypothesized relationship between genetic variation and laterality, finding that in Chinook salmon there is potential for an effect of inbreeding on lateralized morphology, but not in the expected direction.

## 1. Introduction

The brain is responsible for the direction of body movements, decision making and hormone production, which directs somatic growth [1,2], and it is also one of the most costly vertebrate organs to produce and maintain [3]. The expensive-tissue hypothesis previously suggested a trade-off in growth of gut size to compensate for larger brain size [4], but this formulation of somatic trade-offs has been met with some scepticism [5] leading to an extension of this hypothesized relationship, known as the energy trade-off hypothesis. The energy trade-off hypothesis suggests that increases in brain size are associated with corresponding decreases in energy consumption from ‘flexible functions’, such as reproduction, digestion and locomotion [6,7]. The energy trade-off hypothesis, therefore, may be an evolutionary mechanism to explain constraints on brain and body function.

A decrease in size of some expensive tissues (e.g. brain, gut, reproductive tissue) or the reduction in energy consumption of ‘flexible functions’ could allow for an increase in brain size without increasing net metabolic costs. However it is possible that there are other drivers, thus far overlooked, that are responsible for the size of the brain and other organs. Inbreeding, or mating between closely related individuals, will often lead to inbreeding depression: a decrease in an individual's fitness due to increased genetic homozygosity and the expression of recessive deleterious alleles [8,9]. While life-history traits related to fitness may experience the highest inbreeding effects [10], morphological traits can also be significantly impacted. Inbreeding has led to body weight reductions in rainbow trout (*Oncorhynchus mykiss*), where the consequences of inbreeding became more pronounced with increasing age, and resulted in significantly decreased female reproductive fitness (i.e. egg production) [11,12]. Thus, inbreeding effects on body size, which may be a characteristic crucial to some flexible functions, would also be expected to affect brain size with the energetic trade-offs outlined above.

In addition to energetic trade-offs at the whole brain level, inbreeding may also affect differential investment of the right and left brain hemispheres. Differential responses of brain hemispheres, also known as directional asymmetry or lateralization, have been proposed as a mechanism for increased efficiency of neural processing [13,14] and therefore may respond to differing ‘flexible functions’. In many vertebrate species, it has become apparent that the right and left hemispheres of the brain are responsible for different and specific tasks [14–18], and while most studies of laterality have focused on lateralization of behaviour (e.g. [19–22]), there is increasing evidence of the asymmetry or differential contributions of underlying bilateral neural structures that underpin the roots of asymmetry [23,24]. It has been hypothesized [25–28] that there is a link between levels of genetic variation and directional asymmetry in vertebrates but support for this hypothesis remains inconclusive, with some studies finding a positive relationship between asymmetry of meristic characteristics and inbreeding [25] while others found no association [29]. While the hypothesized role of genetic variation in laterality makes intuitive sense, more testing is required, and on other characteristics including mensual (measured) characters, to either refute or support this hypothesis.

The purpose of the current study is to examine effects of genetic variation on potential trade-offs between brain and somatic growth as hypothesized in the energy trade-off framework, as well as inbreeding effects on brain laterality, as both have been postulated to fluctuate with genetic variation [10,11,26,27]. In addition, lateralization and brain growth have shown evidence of being passed on through some, as yet unidentified, heritable component. Artificial selection on turning behaviour (i.e. greater left or right turning preference) in minnows (*Girardinus falcatus*) over five generations results in offspring showing the same turning preferences as the parental fish [30]. In that study, there might be an underlying lateralized brain component leading to the specific lateralized behavioural output. Indeed brain morphology can be inherited from parents, as demonstrated in guppy (*Poecilia reticulata*) offspring, who were artificially selected for large or small brain size [31], and showed brain size comparable to their parental fish overall. Therefore, we would expect that morphological lateralization of the brain would be inherited from generation to generation. By using offspring from previously created lines of Chinook salmon (*Oncorhynchus tshawytscha*) with different levels of inbreeding, assessed as per cent heterozygosity using fin clips from representative fish of the same genetic lines but different cohorts, we are able to begin to test the potential role of inbreeding in both energetic trade-offs as well as in lateralized differences in gross brain morphology, which has not yet been rigorously investigated. Here we hypothesize that the group deemed to have the highest level of inbreeding (‘Very High’) will show the lowest investment into energetic trade-offs (i.e. brain-to-body ratio) and, based on the suggested genetic variation and asymmetry relationship, will show the greatest asymmetry on our measures of laterality.

## 2. Methods

### 2.1. Sample collection

#### 2.1.1. Study species

All measures were collected from seven different crosses of 3 year old Chinook salmon in the fall of 2012–2014 from Yellow Island Aquaculture Ltd (YIAL) (Quadra Island, British Columbia, Canada), where distinct genetic crosses have been created and maintained since the late 1990s [32]. Our seven genetic crosses consisted first of offspring from self-crossed hermaphrodites which originated at YIAL in 2009 as the result of the incomplete sex-reversal of a female broodstock fish (see [33] for further breeding details). Secondly, we used offspring from crosses maintained as YIAL's broodstock; their ‘high performance’ (HHxHH) and ‘low performance’ (LLxLL) purebred lines. These HHxHH and LLxLL lines were created from fish chosen for high or low performance based on gene markers related to growth and survival (see [32] and [34] for detailed breeding information) rather than from crosses specifically designed to test inbreeding effects. We also used offspring from crosses involving a hermaphrodite fish (H_1_ or H_3_) and high performance line (HH) fish (H_1_ x HH and H_3_ x HH); and our final crosses were made up of hybrid performance offspring (HHxLL and LLxHH fish) from crosses of the purebred genetic lines (see [34] for detailed breeding information). The first letters of the notation for all crosses indicate the maternal line and the second letters indicate the paternal line.

Fin clips were collected from fish from each of the above outlined crosses (see table 1; hybrid performance crosses pooled) of Chinook salmon at YIAL at different times and different stages of development. First, fin clips were collected and preserved in June 2009 from offspring of hybrid performance crosses (HHxLL and LLxHH) when fish were approximately seven months post-fertilization. Fin clips were also collected and preserved from fish from hermaphrodite crosses (self-crossed hermaphrodite offspring; hermaphrodite offspring×normal fish crosses) at approximately 1.5 years post-fertilization in April 2011. Finally, in the fall of 2011, fin clips were collected and preserved from sexually mature individuals from purebred crosses (HHxHH and LLxLL), where individuals ranged from 4 to 5 years in age. It should be noted here that fin clips were not collected from the fish that were sampled for brain and body measurements; instead fin clips were collected from fish in the same genetic lines as our study fish; however some samples may represent groups of different cohorts. Therefore, different samples were used for genotyping to infer heterozygosity of our sample groups.

**Table 1.** Heterozygosity (observed, Ho and expected, He) and number of individuals genotyped (*N*) for six groups of captive Chinook salmon.

genetic crosses	*N*	Ho	He
self-crossed hermaphrodite offspring	29	0.456^a^	0.451^a^
Hermie 1 x High 1 and reciprocal cross (H_1_ x HH; HH x H_1_)	27	0.676^ab^	0.660^ab^
Hermie 3 x High 3 and reciprocal cross (T_3_ x HH; HH x H_3_)	27	0.677^ab^	0.619^ab^
CRD purebred (LLxLL)	31	0.765^ab^	0.683^ab^
CRD purebred (HHxHH)	34	0.787^b^	0.766^b^
CRD hybrid (HHxLL and LLxHH)	29	0.835^b^	0.811^b^

Different letters represent significant differences between groups (alpha level = 0.0083).

Finally, prior to all analyses, genetic crosses were separated into groups based on parental lineage to test the hypothesized relationship between laterality and genetic variation. Thus, all offspring derived from self-fertilization (i.e. hermaphrodites) composed the first group, all offspring derived from hermaphrodite×HH (and the reciprocal) crosses made up the second group, all purebred cross fish (both HHxHH and LLxLL) were a third group, and all hybrid performance fish (HHxLL and LLxHH crosses) constituted our fourth group.

#### 2.1.2. Somatic and brain measurements

To address the energy trade-off hypothesis, two absolute somatic measures, brain mass and body mass, were collected from all fish. After sacrifice and prior to brain removal, the weight of all fish was measured on site in kilograms to two decimal places (Marel M1100, Marel, Gardabaer, Iceland). A small section of the head containing the brain was removed from each fish and preserved in a 50 ml Falcon tube (Corning, Inc., https://www.fishersci.com/) containing 30 ml of 10% buffered formalin for 48–72 h. The formalin was removed and the head sections were transported to the laboratory at the University of Windsor where the brains were dissected from the head section and placed in 70% ethanol. Total brain mass, in grams, was obtained in the laboratory using a two decimal standard scale (Ohaus Scout Pro SP202, Ohaus Corporation, New Jersey, USA). To estimate the growth energy invested into brain versus body growth, a brain-to-body ratio measure was obtained using the two absolute measurements of brain and body mass (absolute brain mass (g)/absolute body mass (g)) [35].

**Figure 1.**
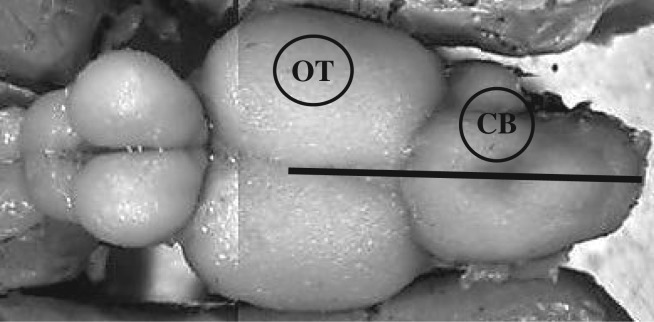
The two salmonid brain regions of interest measured in the present study: the optic tectum (OT) and the cerebellum (CB). The black line indicates where the cerebellum was divided into a right and left hemisphere using the midline of the optic tectum lobes as an anchoring point.

Following brain removal and weighing, dorsal images of all brains were taken with a digital camera (Q-imaging Q1 Cam Fast 1394) connected to a dissecting microscope (Leica L2 10445930). Area and perimeter measurements were collected for the right and left hemispheres of the optic tectum and cerebellum (figure 1) from dorsal brain images using Northern Eclipse imaging software (Empix Inc., http://www.empix.com). Whole brain mounts were used in place of histological sectioning to avoid irregularities of fixation which can cause differential shrinkage of brain regions following tissue dehydration and embedding (e.g. [36]). To obtain left and right hemisphere measurements from the single-lobed cerebellum, this region was superficially bisected. The midline between the right and left optic tecta was used as an anchoring point of reference for the superficial bisection line through the cerebellum (figure 1), as the tectal ventricle and rhombencephalic ventricle within the brain make up the internal midline of the optic tectum lobes, continuing through the cerebellum providing an internal left–right division [37].

#### 2.1.3. Genetic analyses of heterozygosity

DNA was extracted from fin clips following an automated plate-based extraction protocol [38]. Individual genotypes were determined through polymerase chain reactions (PCR) using 10 previously described microsatellite loci, specifically OtsG68, OtsG432, OtsG78b [39], RT212, RT36 [40], Ots 211, Ots213 [41], Ots1 [42], Ots107 [43] and Omy325 [44]. All primers were fluorescently dye-labelled and thus PCR products could be visualized using a LiCor 4300 DNA analyzer (LiCor Biosciences, Inc.). Fragment sizes (alleles) were then scored using GENE IMAGIR 4.05 software (Scanalytics Inc.).

Using the heterozygosity estimates we were able to assign each of our previously organized genetic crosses into a ‘level of inbreeding’ group, ranging from low to very high, allowing us to more readily hypothesize about where each group may fall according to the genetic variation and laterality hypothesis. The four ‘levels’ defined from the analyses of heterozygosity were as follows. The ‘Very High’ inbreeding level label was given to our offspring derived from self-fertilization (i.e. from self-crossed hermaphrodites); these were the fish with the lowest average genetic variation (average heterozygosity: 46%). The label of ‘High’ inbreeding (average heterozygosity: 68%) was for those fish whose parentage consisted of a hermaphrodite parent (H_1_ or H_3_) and normal stock (HH) fish (denoted as H_1_ x HH or H_3_ x HH crosses, and the reciprocals; see table 1). Fish from purebred crosses (HHxHH and LLXLL) (average heterozygosity: 77%) were given the label of ‘Medium’ inbreeding level, and the ‘Low’ inbreeding level label was given to the hybrid performance offspring (average heterozygosity: 84%).

### 2.2. Statistical analyses

#### 2.2.1. Somatic and brain measurements

Prior to analyses, assumptions of normality, homogeneity of variance and lack of outliers were assessed. For assumptions to be met, 15 cases were removed due to incomplete dissection and damage to key brain regions, leaving us with a total sample size of 118 fish.

The brain-to-body ratio was used as a measure of the energy trade-off hypothesis, calculated using the formula: brain mass (g)/body mass (g). Differences between the inbreeding level groups were investigated using a univariate ANOVA. Tukey's *post hoc* analyses provided clarification of significant effects of inbreeding level.

Left and right hemisphere measurements of perimeter and area were collected from dorsal images of all brains extracted and were used to obtain the ‘laterality index’, LI = (L − R)/(L + R), where ‘L’ indicates the left side measurements and ‘R’ indicates right side measurements [24]. This formula allows for a determination of *side dominance* and a consideration of asymmetry of each region independent of overall brain size. Positive values (from 0 to +1) are indicative of greater left hemisphere size whereas negative values (from 0 to −1) are indicative of greater right hemisphere size. In addition, the absolute (unsigned) value of the LI was taken (i.e. | LI |) as a measure of the strength of asymmetry irrespective of direction [45,46]. Because of a strong correlation between the laterality index and absolute index values of the optic tectum (*r* = 0.215, *p* = 0.019), and between laterality index and absolute index values of the cerebellum (*r* = 0.531, *p* < 0.001)—but no correlation between the laterality and absolute index values across regions—two separate MANOVAs were run: one for the laterality index measures and one for the absolute index measures. Analyses were carried out in this way as results are more reliable when the dependent variables being investigated in a MANOVA are not, themselves, related [47,48]. Because two separate tests were run we used a Bonferroni corrected alpha value of *p* = 0.025 (0.05/2) for our brain morphology results. Perimeter values are reported here as patterns for differences between groups were similar with respect to area and perimeter measurements.

#### 2.2.2. Heterozygosity

Individuals that were genotyped at fewer than 6 loci were removed from subsequent analyses. All genetic analyses therefore included 27–34 individuals for each of the six groups (table 1). We tested for significant deviations from Hardy–Weinberg equilibrium (HWE) at all loci using GenePop v. 4.2 [49]. We also tested for significant linkage disequilibrium using GenePop v. 4.2 [49], with an adjusted alpha level of 0.005 (*p* = 0.05/10) given multiple pairwise comparisons among the 10 loci. Mean observed (H_O_) and expected (H_E_) heterozygosity across all loci were calculated using GenAlEx v. 6.5 [50]. Heterozygosity estimates were compared among groups using the Kruskal–Wallis test, and if significant differences were detected then Tukey's *post hoc* tests were performed. Given that we conducted multiple comparisons among the six groups, we chose an adjusted alpha level of 0.0083 (*p* = 0.05/6) for the analyses.

## 3. Results

### 3.1. Heterozygosity estimates

No loci showed significant deviations from HWE in any of the six groups, and no pairs of loci showed significant linkage disequilibrium (*p* < 0.005) in more than two of the six groups. Observed heterozygosity ranged from 45.6 to 83.5%, and was significantly different between groups (table 1; *p* = 0.0005). *Post hoc* tests revealed that self-crossed hermaphrodite offspring experienced statistically significantly lower heterozygosity compared to both HHxHH (*p* = 0.006) and hybrid groups (*p* = 0.0003). Expected heterozygosity was also statistically significantly different between the groups (table 1; *p* < 0.001), where self-crossed hermaphrodite offspring showed significantly lower expected heterozygosity relative to both HHxHH and hybrid groups (*p* values < 0.001).

### 3.2. Somatic trade-offs

**Figure 2.**
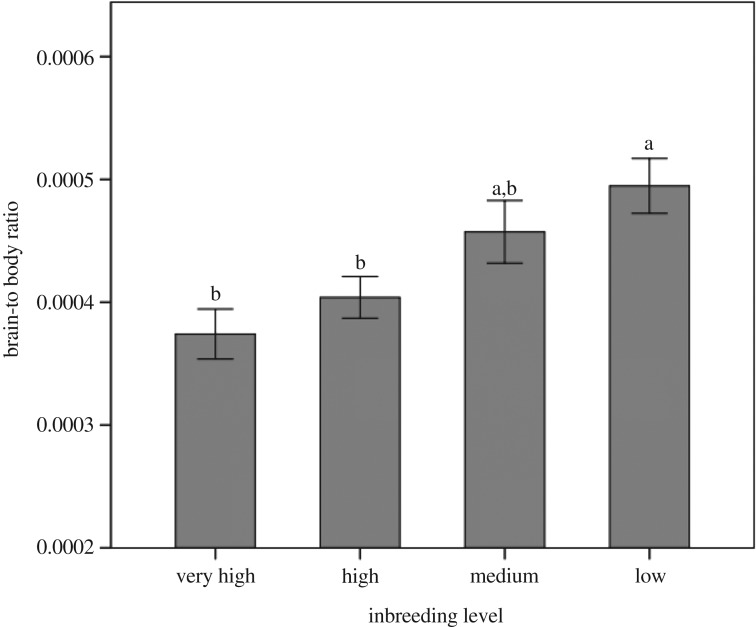
As a measure of the energy trade-off hypothesis, the mean brain-to-body ratio values across inbreeding levels indicate that those fish with the lowest inbreeding level, and thus highest per cent of heterozygosity, show the greatest investment into brain mass when body mass is taken into account. Error bars represent mean ± 1 standard error.

There was a statistically significant effect of inbreeding level on the brain-to-body ratio measure, indicating differential investment of growth energy into the brain versus the body (*F* (3, 114) = 5.140, *p* = 0.002 (figure 2)). The Low inbreeding level group showed an overall greater investment into brain growth when body growth was taken into account, whereas the Very High inbreeding group showed the lowest brain versus body investment. A Tukey's *post hoc* analysis revealed that these differences were greatest between the Low and Very High (*p* = 0.004), and Low and High (*p* = 0.029) groups (figure 2).

### 3.3. Laterality measures

**Figure 3.**
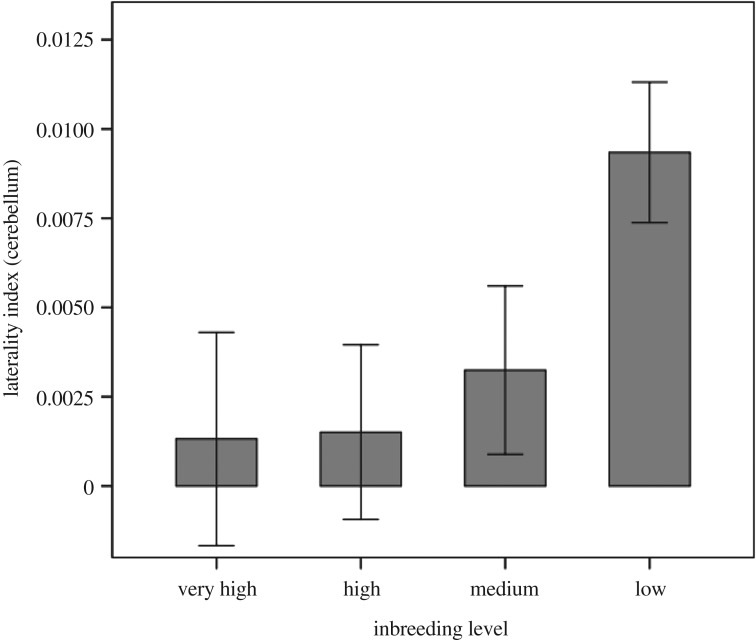
Representation of the laterality index of the cerebellum. Note that the values are all positive, indicating a larger left side of the cerebellum in fish of all inbreeding levels. Error bars represent mean ± 1 standard error.

Multivariate tests indicated that there was no effect of inbreeding level on the absolute asymmetry values (Wilks' lambda, *Λ* = 0.950, *F* (6, 226) = 0.988, *p* = 0.434). Multivariate tests on the laterality index values showed that, while not significant, the effect of inbreeding level was close to our threshold for statistical significance (*Λ* = 0.890, *F* (6, 226) = 2.261, *p* = 0.039). Despite the overall non-significant effect for the laterality index (at our corrected alpha value) the between-subjects effects of inbreeding level were examined. These tests showed that there was no significant effect of inbreeding level on the directionality of the optic tectum (*F* (3, 114) = 1.566, *p* = 0.202) but there was an effect on the cerebellum (*F* (3, 114) = 3.005, *p* = 0.033), and while all four groups showed a larger left cerebellar hemisphere as indicated by the positive laterality index values (figure 3), the Low inbreeding level had the highest laterality index (see table 2 for all values). *Post hoc* tests were not statistically significant between all groups and only the difference between the Low and High inbreeding groups approached marginal significance (*p* = 0.081) (figure 3), but the differences appear to be driven mainly by the Low inbreeding level group.

**Table 2.** Mean (*M*), standard error (s.e.) and confidence intervals (95% CI) for the effect of inbreeding level on four measures of morphology.

morphology measure	inbreeding level	*M*	s.e.	95% CI (lower, upper)
optic tectum, laterality index	very high	−0.00297	0.00415	−0.01170, 0.00575
	high	0.00822	0.00520	−0.00255, 0.01898
	medium	0.01305	0.00408	−0.00470, 0.02139
	low	0.00579	0.00446	−0.00320, 0.01478
optic tectum, absolute index	very high	0.01625	0.00176	0.01256, 0.01993
	high	0.02149	0.00315	0.01497, 0.02801
	medium	0.02053	0.00282	0.01476, 0.02631
	low	0.02397	0.00276	0.01841, 0.02953
cerebellum, laterality index	very high	0.00132	0.00299	−0.00496, 0.00760
	high	0.00151	0.00245	−0.00355, 0.00657
	medium	0.00324	0.00236	−0.00158, 0.00806
	low	0.00934	0.00197	0.00538, 0.01331
cerebellum, absolute index	very high	0.01091	0.00155	0.00765, 0.01417
	high	0.01052	0.00113	0.00820, 0.01285
	medium	0.00998	0.00158	0.00675, 0.01320
	low	0.01305	0.00141	0.01022, 0.01589

## 4. Discussion

### 4.1. Somatic trade-offs

The energy trade-off hypothesis has not explicitly been investigated with reference to genetic variation and its effects on potential differences between the brain and the body, but our results begin to suggest that there may be differential effects of genetic variation on a brain-to-body trade-off measure, although this would need to be confirmed with a breeding design specifically set up to test inbreeding effects. Other work on the energy trade-off hypothesis has examined the relationship between the brain and the gonads in bats [51], pectoral muscle mass in multiple bird species [6], the number of offspring produced in guppies [31], and egg size and duration of parental care in cichlids, which both showed a positive correlation with brain size [7]. In our study, when the brain and body mass were considered together as a reflection of the energy trade-off hypothesis we saw that the Very High inbreeding group showed the lowest brain-to-body ratio, and differed significantly from the most genetically variable group (Low inbreeding) that showed the highest ratio. This relative measure of brain to body mass is a reliable proxy for investigating patterns of somatic investment [35] and here we are seeing divergence in brain size as a function of body size between groups of differing heterozygosities [52], based on our grouping system. Because there may be other uncontrolled for genetic differences between our groups, it is possible that differences in heterozygosity are not solely responsible for the differences observed so a follow-up study could use a controlled breeding design. A higher relative investment in brain size coupled with a higher level of heterozygosity *may* be a potent combination for overall fitness and survival given that genetic history has shown important influences on body size (e.g. [11,12,53,54]) and on gonadosomatic index (i.e. a trade-off between body size and gonad size) [55] in fish, and that inbred (i.e. low heterozygosity) individuals have, overall, shown decreases in growth, fitness and survival rates [8,56]. Enhanced investment in brain over body, then, may indicate enhanced sensory or behavioural abilities [31]. Fish in aquaculture facilities are often highly inbred (e.g. [57]) and optimized for high growth rates (e.g. [58]) but when aquaculture fish are released into the wild for restocking purposes they often experience high levels of mortality due to predation (e.g. [59,60]). One option to enhance post-release survival when restocking may be to focus on increasing genetic diversity in offspring to enhance relative brain size and, perhaps, cognitive abilities since our results showed a potential linkage between heterozygosity and relative brain size.

### 4.2. Genetic effects on laterality

As a test of the hypothesized relationship between genetic variation and measured asymmetry, our results, like others [25,29], showed mixed support. Of the three brain regions measured, only one region—the cerebellum—showed any indication of differences of measured laterality between groups. Interestingly, it was *not* the Very High inbreeding level group made up of offspring of self-crossed hermaphrodites that showed the greatest asymmetric differentiation. The genetic variation/asymmetry hypothesis suggests that organisms with the *highest* inbreeding levels would have correspondingly high measures of asymmetry, yet here the only significant result of laterality indicates that our group with the *lowest* inbreeding level had the highest measured morphological asymmetry since the Low inbreeding group was driving significant effects seen. Granted, all fish in the present sample from whom morphological measures were obtained are from farmed crosses and are likely to experience more inbreeding than their wild conspecifics, but it seems that a higher degree of inbreeding, or at least a lower degree of heterozygosity, is not completely sufficient to predict greater values of measured asymmetry on a morphological measure, at least in Chinook salmon.

Previous studies investigating brain differences in fish have done so using brain size as a function of environmental rearing conditions generally focusing on each brain region as a whole [3,50,61–63]. Fewer studies, however, have actually investigated asymmetric differences of brain regions and, when looking at neuroanatomy, have been more likely to focus on smaller neuroanatomical features like the habenular nuclei (see [64] for review). Ours is one of the first studies to investigate the morphological differences between hemispheres of the salmonid brain, whose growth is continuous throughout the life cycle [36,65] responding to both external stimuli, such as environmental rearing conditions, and internal physiological status. Previous work has shown that larger overall brain size is related to increased cognitive ability in a fish species, *Poecilia reticulata* [31], suggesting perhaps a greater number of or larger neurons within the brain. Differential growth of the left versus right hemispheres in a fish, then, may be related to greater reliance on and use of one hemisphere of a given region due to increased dependence for stimulus processing. Having a lateralized brain has been hypothesized to be beneficial ([28] and references therein) but there has been little connection to how this benefit might correlate with or be explained by larger brain regions. In fish, the cerebellum is responsible for motor control, muscle coordination and general movement [66]; therefore if a greater number of synaptic connections, or larger or more numerous neurons are found delegated to the left hemisphere, for example, fish may show a propensity for and efficiency of escape, random turns, or general movement in a rightward direction. However the connection between motor asymmetries and hemispheric differences of the cerebellum has yet to be rigorously investigated.

## 5. Conclusion

Here we have presented one of the first studies to use crosses with different levels of heterozygosity, likely caused by inbreeding to examine the potential effect of genetic variation on somatic trade-offs and, using measures of lateralization of brain morphology, to assess the hypothesis outlining a relationship between genetic variation and measured asymmetry. While our lines were not specifically bred to control for inbreeding and there may be other genetic differences involved in the response, our results suggest that a reduction in genetic variation does lead to a reduced brain-to-body ratio. Our study is only beginning to examine patterns that may exist with respect to genetic variation but because we did not carry out a specific and controlled inbreeding design we can only suggest potential effects of genetic variation. In the future, more controlled studies of inbreeding will need to be carried out to get at the true effect that genetic variation has on somatic trade-offs and lateralized brain morphology. Investigating through controlled breeding how genetic makeup may influence the division of energy to certain tissues could hold potential for aquaculture facilities and restocking programmes aiming to ensure the healthiest fish possible with the greatest chance of survival [55]. In investigating differences in laterality as an effect of our grouping variables of ‘inbreeding level’, we found some evidence that greater heterozygosity may lead to greater laterality and our study is the first, to our knowledge, to address this hypothesis in detail with respect to brain hemisphere differences. Further work on this hypothesis must be done to gain a better understanding of how genetic variation may affect measures of lateralization. Studies of lateralized morphology and behaviour to date have largely left out the component of genetic background of the study organisms but moving forward studies wishing to test the genetic variation and laterality hypothesis must incorporate breeding designs necessary to test inbreeding effects to truly understand the nature of the suggested relationship. Based on the inbreeding level groups estimated herein, we have shown that there may be effects of genetic background, yet not in the manner that has been previously hypothesized.

## Data accessibility

Data are available at http://dx.doi.org/10.5061/dryad.rg22r [67].
